# The second international genomic medicine conference (2^nd^ IGMC, 2013): brief report and outcome

**DOI:** 10.1186/1755-8794-8-S1-S1

**Published:** 2015-01-15

**Authors:** Muhammad M Abu-Elmagd, Mohammed H Al-Qahtani

**Affiliations:** 1 Center of Excellence in Genomic Medicine Research (CEGMR), King Abdulaziz University, P.O. Box: 80216, Jeddah 21589, Kingdom of Saudi Arabia; 2 KACST Centre of Innovation in Personalized Medicine (CIPM), King Abdulaziz University, P.O. Box: 80216, Jeddah 21589, Kingdom of Saudi Arabia; 3 Zoology Department, Faculty of Science, Minia University, El-Minia, P.O. Box 61519, Egypt

## Introduction

Centre of Excellence in Genomic Medicine Research (CEGMR, at King Abdulaziz University (KAU) [1], is a leading centre in the field of medical genomics research in the Western province of the Kingdom of Saudi Arabia. It was founded in 2007 with the aim of addressing important health concerns nationally and internationally. One approach the CEGMR implemented to help achieve this aim was to host an annual international conference. An initiative considered to be an excellent opportunity to interact with the international science community, discuss and exchange ideas, and set up collaboration through networking. In November 2012 at KAU, CEGMR represented by its Director (Professor Mohammed Al-Qahtani), Deputy Directors (Professor Adel Abuzenadah and Dr Adeel Chaudhary), the supervisor of the continuing education and postgraduate studies (Professor Mamdooh Gari), and all research and technical staff organized its first International Genomic Medicine Conference (1^st^ IGMC). In November 2013, CEGMR continued its leading role in genomics and organized its 2^nd^ IGMC. The conference was well received and attended by 505 international and national participants. Among the international participants was Professor Erwin Neher, a Nobel laureate with Bert Sakmann in 1991 in Physiology or Medicine, who gave the keynote talk on the first day of the conference on his discovery of “The function of single ion channels in cells” with notes on their function and role in disease. His presence and talk was an excellent opportunity for junior and senior national researchers in Saudi Arabia to meet and learn from a scientific pioneer.

The 2^nd^ IGMC scientific committee led by Dr Muhammad Abu-Elmagd worked tirelessly hard and managed, for the first time ever for the conference, to publish 98 IGMC abstracts and more than 24 peer-reviewed full articles in two separate special supplements in a high impact factor (IF) journal in the field, the BMC Genomics (IF: 4.04) [2]. These two supplements mark an unprecedented success for the CEGMR and the IGMC.

## Summary of the oral presentations

Oral presentation sessions started with a talk given by Dr Abdulaziz Al-Swailem from KCAST who elaborated on Saudi national science, technology and innovation plans towards knowledge based economy. After Dr Al-Swailem’s presentation, the conference participants enjoyed attending a series of 17 two days excellent talks presented by Thomson Reuters’s ranked highly distinguished scientists who came from various institutions around the globe to deliver presentations in different topics. The latter mainly covered five genomics and genomic-related areas including oncogenomics, genomics and environment interactions, systems biology and computational genomics, immunogenomics, and omics of cell signalling and reproductive biology (2^nd^ IGMC program, Table 1). The conference was also a good opportunity for both junior and senior speakers from Saudi Arabia to deliver 10 excellent presentations. These speakers were from academic, health and research institutions in different regions of Saudi Arabia. The organising committee also gave the chance to the industrial sector to give a talk and this was delivered by Life Technologies representative Dr. Barry Merriman**.** Abstracts of all oral presentations are now published in BMC Genomics special supplement [2].

**Table 1:** The 2^nd^ IGMC program

Day One Sunday 24^th^ November 2013	
08:00-09:00	Registration

09:00-09:15	Opening Ceremony

09:15-09:30	KAU Endowment (Wagf) Introduction

09:30-09:50	**Dr. Abdulaziz Al-Swailem** “Saudi national science, technology and innovation plan towards knowledge based economy”

**Session (1): ONCOGENOMICS**	

**Chairpersons:** ***Prof. Paolo Sassone-Corsi & Prof. Adnan Merdad***	

09:50-10:20	**Prof. Jerry Shay** “Identification of small molecule inhibitors that target truncated adenomatous polyposis coli (APC) in colon cancer”

10:20-10:50	**Prof. Albert Joseph Fornace Jr.** “Addition of metabolomics to the omics spectrum for cancer biomarker development – demonstration with the KrasG12D mouse model for pancreatic carcinoma”

10:50-11:05	Coffee Break

**Chairpersons:** ***Prof. Emmanouil Dermitzakis & Dr. Abdulaziz Al-Swailem***	

11:05-11:35	**Prof. Angel Carracedo** "Pharmacogenomics of CRC treatments"

11:35-11:55	**Dr. Abdelbaset Buhmeida** “Molecular characterization of colorectal carcinoma of Saudi Arabian patients and identification of predictors of disease outcome”

11:55-12:15	**Dr. Brian Meyer** “Application of next generation sequencing in oncology”

12:15-12:35	**Dr. Ashraf Dallol** "The methylome of breast cancer in Saudi Arabia "

12:35-13:30	Prayers, Lunch & Poster Sessions

**Session (2): FRONTIERS OF GENOMICS-ENVIRONMENT INTERACTIONS**	

**Chairpersons:** ***Prof. Eberhard Nieschlag & Dr. Sultan Al-Sedairy***	

13:30-14:30	**KEYNOTE SPEECH** **PROF. ERWIN NEHER (Nobel Prize Laureate)** “Ion channels: their discovery, their function, and their role in disease”

14:30-14:50	**Dr. Ibrahim Abdulkareem** “How does Arab genome different from other genomes”

14:50-15:45	Coffee Break & Poster Sessions

**Chairpersons:** ***Prof. Albert Fornace Jr & Dr. Mohammed Al-Balwi***	

15:45-16:15	**Prof. Paolo Sassone-Corsi** “Epigenetics and metabolism: The clock connection”

16:15-16:45	**Prof. Frank Hu** “Nutritional genomics and metabolomics in obesity and type 2 diabetes”

16:45-17:05	**Prof. Valerio Orlando** “Epigenetic control of cell identity and plasticity”

**Day Two** **Monday25^th^ November 2013**	

08:30-09:00	Reception, Coffee & Poster Sessions

**Session (1): SYSTEMS BIOLOGY & COMPUTATIONAL GENOMICS**	

**Chairpersons:** ***Prof. Sudhir Kumar & Dr. Adeel Chaudhary***	

09:00-09:30	**Prof. Emmanouil Dermitzakis** “Understanding the phenotypic impact of non-coding sequence variation”

09:30-10:00	**Prof. Sudhir Kumar** “Next generation methods and tools for timing the evolution of species and strains”

10:00-10:30	**Prof. Adam Godzik** “Structural systems biology – from bacterial to cancer networks”

10:30-10:50	**Dr. Barry Merriman** “Solving genetic disease at the population scale”

10:50 – 11:05	**Mr. Ayman Abuelela** “Sialyl Lewis X/A-forming glycosyl transferases controlled by prometastatic and cell stemness transcription factors: meta-analysis of ENCODE ChIP-Seq data."

11:05-11:20	Coffee Break

**Session (2): IMMUNOGENOMICS**	

**Chairpersons:** ***Prof. Nobutaka Hirokawa & Dr. Aayed Robiaan Al-Qahtani***	

11:20-11:50	**Prof. FooYew Liew** “The Immunogenetics of rheumatoid arthritis”

11:50-12:20	**Prof. Ali Mobasheri** “Biomarkers of osteoarthritis: translating information from *in vitro* culture systems to human patients”

12:20 – 12:30	**Dr. Hani Choudhry** “Unlocking the complexity of hypoxia non-coding transcriptome landscape of breast cancer”

12:30 - 12:40	**Dr. Nouf Laqtom** “The regulation and function of miR-199-3p in CMV infections”

12:40-13:40	Prayer & Lunch

**Session (3): THE OMICS OF CELL SIGNALING & REPRODUCTIVE BIOLOGY**	

**Chairpersons:** ***Prof. Jerry Shay & Dr. Mohammed Al-Qahtani***	

13:40-14:10	**Prof. Norbert Perrimon** “Signaling in time and space”

14:10-14:40	**Prof. Nobutaka Hirokawa** “Intracellular transport and molecular motors, KIFs”

14:40-15:10	**Prof. Stephen Scherer** “Autism and related neurodevelopmental disorders: so many genes involved”

15:10-15:40	**Prof. Eric Olson** “The molecules and mechanisms of muscle development, disease and regeneration”

15:40-16:00	Coffee Break

**Chairpersons:** ***Prof. Ali Mobasheri & Prof. Jaudah Al-Maghrabi***	

16:00-16:20	**Dr. Muhammad Abu-Elmagd** “Sprouty2 mediated tuning of signaling is essential for somite myogenesis“

16:20-16:50	**Prof. Ashok Agarwal** “Impact of sperm chromatin damage on natural fertility and art success: evidence based medicine”

16:50-17:20	**Prof. Eberhard Nieschlag** “Genetics of male infertility”

17:20-17:50	Closing Remarks & Ceremony

**Day Three** **Tuesday 26^th^ November 2013**	

**WORKSHOPS**	

08:00-17:00	**Application of Molecular Cytogenetics (Dr. Ibtessam Ramzi Hussein)**

08:00-17:00	**High Throughput DNA Sequencing (Dr. Ashraf Dallol)**

08:00-17:00	**Application of Molecular Genetics (Dr. Muhammad Imran)**

08:00-17:00	**Application of Microarrays (Dr. Hans Schulten)**

08:00-17:00	**Flow Cytometry in Oncology (Dr. Farid Ahmed)**

**Day Four** **Wednesday 27^th^ November 2013**	

**WORKSHOPS**	

08:00-16:00	**Power of Bioinformatics (Dr. Sajjad Karim)**

## Peer-reviewed articles

Among all abstracts submitted to the 2^nd^ IGMC, 32 were selected by the conference scientific committee to be re-submitted as full articles. All authors who were invited to submit their work as full articles responded positively. These articles were sent to a carefully-selected high calibre international and national scientists in the field for reviewing. BMC Genomics editorial board accepted 23 out of the 32 articles which included 17 research papers and 6 reviews. All articles have undergone the BMC journal's standard peer review process for supplements.

## Poster presentations

The second IGMC allocated three poster sessions during which 73 posters covering different aspects of genomics were presented. The Director of the CEGMR Dr Mohammed Al-Qahtani announced the winners of the best posters presented during the conference. The first winner was Mrs Manal Al-Otaibi who presented a poster titled ''Sunitinib effectively reduces clonogenic acute myeloid leukemia cells *in vitro*'', the second winner was Mrs Shilu Mathew who presented a poster titled ''Identification of frequent MTNR1B methylation in breast cancer following the application of high-throughput methylome analysis'' and finally the third winner was Mr. Nasser Elthawary who presented a poster titled: ''Azoospermia factor microdeletions: common tag STSs in infertile men with azoospermia and severe oligospermia from Egypt''.

## Professor Erwin Neher remarks on the second IGMC

The 2^nd^ IGMC had the privilege of hosting Professor Erwin Neher who delivered an inspiring talk about his discovery of the patch clamp techniques. Professor Neher was asked by the organizing committee to write some comments about his impression of the 2^nd^ IGMC. The following is what he wrote:

''Genomics is not my field of research. As a scientist, working on cellular function, I observe, of course, progress in sequencing technology and read about many of the insights, which emerge from the huge data sets now available through deep sequencing. However, participating in a meeting dedicated to the medical applications of these new technologies is another type of experience. One could feel the spirit of optimism regarding new therapies for cancer, congenital diseases, and autoimmune disorders. One could listen to brilliant accounts, delivered by international leaders in the respective fields. One could find out details on the latest technology in the exhibitions and speak with the developers of sequencing technology – who, to my delight, were very interested in the patch clamp technique. All this happened in a lush congress centre, offering all the services and amenities, which turn a conference into a pleasant experience. Last not least we had the privilege to enjoy the generous hospitality of our Saudi hosts. This culminated in a memorable visit at night to the estate of Professor Mohammed Al-Qahtani, The Director of Centre of Excellence in Genomics Medicine Research (CEGMR), where we not only enjoyed tasty and spicy Saudi food, but also had a chance to inspect his remarkable facility for keeping desert wildlife.''

## List of abbreviations

APC: Adenomatous Polyposis Coli; BMC: BioMed Central; CEGMR: Center of Excellence in Genomic Medicine Research; ChIP-Seq: Chromatin Immuno-precipitation Sequencing; CIPM: Centre of Innovation in Personalized Medicine; CMV: Cytomegalovirus; CRC: Colorectal Cancer; ENCODE: Encyclopedia of DNA Elements; IF: Impact Factor; IGB: Integrated Gulf Biosystem; IGMC: International Genomic Medicine Conference; KAU: King Abdulaziz University; KACST: King Abdulaziz City for Science and Technology; KIFs: Kinesin superfamily proteins; Kras: Kirsten rat sarcoma viral oncogene homolog; KSA: Kingdom of Saudi Arabia; MOH: Ministry Of Higher Education; MTNR1B: Melatonin Receptor 1B; STSs: Sequence-Tagged Sites

## Disclosures

The authors disclose no competing interests.

## Declarations

Publication charges for this article were funded by the Centre of Excellence in Genomic Medicine Research, King Abdulaziz University, Jeddah, Kingdom of Saudi Arabia.

This article has been published as part of *BMC Medical Genomics* Volume 8 Supplement 1, 2015: Selected articles from the 2nd International Genomic Medical Conference (IGMC 2013): Medical Genomics. The full contents of the supplement are available online at http://www.biomedcentral.com/bmcmedgenomics/supplements/8/S1
